# 

**Published:** 1896-02

**Authors:** 


### CONTENTS.

COMMUNICATIONS.

Four Requisites in Development ........ 53
Dental Examining Boards in the United States and Preliminary Education ........ 59
Southern Dental Association ........ 67
Materia Medica and Therapeutics ........ 91

SELECTIONS.

Surgery Without Pain ........ 93
The Use and Abuse of the Brain ........ 94
Separation of Teeth ........ 95
Liquid Air ........ 96
Heredity ........ 96
New Method of Administering Chloroform ........ 97
Manners of Doctors ........ 98
Guaiacol as a Local Anæsthetic ........ 99
The House We Live in ........ 99
Overdone Sterilization ........ 100
Over Supply of Diminished Demand ........ 100
How to Avoid Colds ........ 101
Poisonous Action of the Aqueous Vapor of Expired Air ........ 102
Municipal Housekeeping in Paris ........ 102
Children's Food ........ 102
To Open the Eustachian Tube ........ 103
A Mixture ........ 103

EDITORIAL.

A Correspondent ........ 103

